# Nerve trauma of the lower extremity: evaluation of 60,422 leg injured patients from the TraumaRegister DGU® between 2002 and 2015

**DOI:** 10.1186/s13049-018-0502-5

**Published:** 2018-05-15

**Authors:** Torge Huckhagel, Jakob Nüchtern, Jan Regelsberger, Mathias Gelderblom, Rolf Lefering, the TraumaRegister DGU®

**Affiliations:** 1 Department of Neurosurgery, University Medical Center Hamburg-Eppendorf, Martinistraße 52, 20246 Hamburg, Germany; 2 Department of Trauma, Hand and Reconstructive Surgery, University Medical Center Hamburg-Eppendorf, Hamburg, Germany; 3 Department of Neurology, University Medical Center Hamburg-Eppendorf, Hamburg, Germany; 4 Institute for Research in Operative Medicine (IFOM), University of Witten / Herdecke, Cologne, Germany; 5 Committee on Emergency Medicine, Intensive Care and Trauma Management (Sektion NIS) of the German Trauma Society (DGU), Berlin, Germany

**Keywords:** Peripheral nervous system, Nerve, Lower extremity, Lower limb, Leg, Damage, Injury, Epidemiology

## Abstract

**Background:**

Nerve lesions are well known reasons for reduced functional capacity and diminished quality of life. By now only a few epidemiological studies focus on lower extremity trauma related nerve injuries. This study reveals frequency and characteristics of nerve damages in patients with leg trauma in the European context.

**Methods:**

Sixty thousand four hundred twenty-two significant limb trauma cases were derived from the TraumaRegister DGU® between 2002 and 2015. The TR-DGU is a multi- centre database of severely injured patients. We compared patients with additional nerve injury to those with intact neural structures for demographic data, trauma mechanisms, concomitant injuries, treatment and outcome parameters.

**Results:**

Approximately 1,8% of patients with injured lower extremities suffer from additional nerve trauma. These patients were younger (mean age 38,1 y) and more likely of male sex (80%) compared to the patients without nerve injury (mean age 46,7 y; 68,4% male). This study suggests the peroneal nerve to be the most frequently involved neural structure (50,9%). Patients with concomitant nerve lesions generally required a longer hospital stay and exhibited a higher rate for subsequent rehabilitation. Peripheral nerve damage was mainly a consequence of motorbike (31,2%) and car accidents (30,7%), whereas leg trauma without nerve lesion most frequently resulted from car collisions (29,6%) and falls (29,8%).

**Conclusion:**

Despite of its low frequency nerve injury remains a main cause for reduced functional capacity and induces high socioeconomic expenditures due to prolonged rehabilitation and absenteeism of the mostly young trauma victims. Further research is necessary to get insight into management and long term outcome of peripheral nerve injuries.

## Background

Trauma of the upper and lower extremity is often combined with additional peripheral nerve injury (PNI). In a single-center study 920 out of 5721 patients with injured extremities suffered from associated nerve lesions with the need of nerve specific surgical procedures like suture or grafting [1]. In another series PNI could be detected by clinical examination and electrodiagnostics in 34% of patients with traumatic brain injury during the postacute care [2]. According to an investigation from the Iran National Trauma Registry database (*n* = 16,753 patients) 1,3% of all trauma victims suffer from PNI, but this proportion may be dependent from the socioeconomic context [3]. The situation in European countries with distinct conditions and regulations might be different and has not been elucidated in detail by now despite of its high therapeutic relevance and diminished quality of life resulting from reduced functional capacity, pain (e.g. from neuroma) and subsequent psychological impairment associated with peripheral nerve conditions [4–6]. The objective of this study is to clarify the frequency and characteristics of nerve trauma in a large European cohort of 60,422 leg injured patients. The provided data may guide the clinician to detect nerve lesions in trauma victims presenting with certain risk factors earlier which could eventually improve their functional outcome.

## Methods

All epidemiological data presented in this investigation were retrieved from the TraumaRegister DGU® (TR-DGU). The present study is in line with the publication guidelines of the TraumaRegister DGU® and registered as TR-DGU project ID 2016–014.

We evaluated 60,422 lower extremity injury cases from the above mentioned registry encoded between 2002 and 2015. A detailed comparison between leg trauma patients without recorded nerve damage (control group) and those with lower limb peripheral nerve injuries (PNI group) was made. Injuries of the lower extremity can be identified by an Abbreviated Injury Score (AIS) beginning with number 8. Only lesions with a minimum AIS grade of 2 were considered. By implementing this specification the inclusion of patients presenting only minor lesions of the legs was prevented. All involved trauma cases were registered in Germany (87,6%) or other European countries contributing to the TR-DGU. Additional detailed information about the registry itself and all currently participating countries is available at the TR-DGU website (www.traumaregister-dgu.de). We excluded data from non-European nations, because the determined purpose of this study is to provide information related to lower extremity nerve damage in the European socioeconomic context. All assessed cases suffered from at least one injury rated with a maximum AIS of 3 or higher. This limitation ensured that every involved leg trauma patient sustained at least one major lesion. Furthermore we suspended all registered admissions from analysis which were transferred to another hospital in the early posttraumatic period up to 48 h to eliminate systematic double assessment. Sixty thousand four hundred twenty-two individuals complied with the requirements and therefore could be included in this clinical investigation. All patients were evaluated for age, gender, trauma mechanism, injury severity scale (ISS), peripheral nervous system involvement, further involved anatomical structures (soft tissue, bones, joints, vessels) and severely affected body segments (head, thorax, abdomen, extremity). Due to the coding practice of the TR-DGU leg amputations were not classified as nerve lesions. A relevant injury to a specific body segment was defined as AIS > = 3 points. Additionally we report on nerve focused surgical procedures, ICU treatment, duration of hospital stay and Glasgow Outcome Scale (GOS) after discharge from the hospital. Injuries of the peripheral nervous system were classified into femoral, sciatic, tibial, peroneal and toe nerve lesions. Variables were analyzed using descriptive statistics (percentages and frequencies) and central tendency measures (mean and median). For small subgroups and pivotal findings the 95% confidence interval (CI) is additionally provided in parentheses.

## Results

### Epidemiology and mechanisms of lower extremity nerve injury

**Fig. 1:**
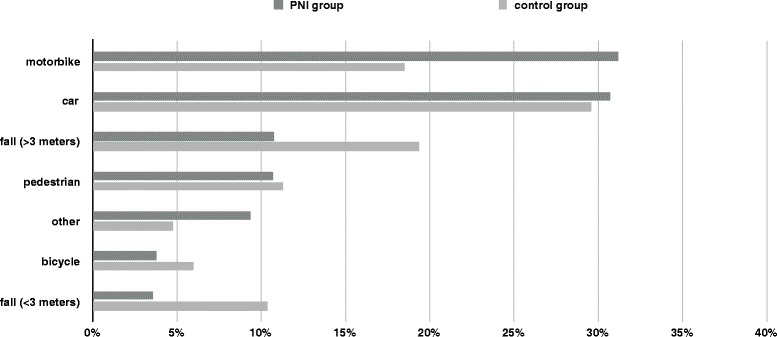
Trauma mechanisms_control_PNI Appendix: Control group = leg trauma patients without recorded nerve injury; PNI group = leg trauma patients with accompanying nerve injury

Among 60,422 patients with significant lower extremity injury we identified 1058 individuals with additional peripheral nerve injury. The mean age of PNI patients was 38,1 years, whereas the mean age of the control group was 46,7 years. On the average leg trauma patients with additional nerve injury were more than eight years younger than their counterparts without peripheral nervous system involvement. Further analysis with subdivision into age bands was performed for both cohorts and is given in Table 1. 86,1% of all PNI patients were between 16 and 59 years old, whereas only 67,5% of the control group belonged to this category. 80% of PNI individuals were male in contrast to 68,4% in the control group. Recorded PNI comprised 113 femoral, 269 sciatic, 204 tibial, 538 peroneal and 11 toe nerve lesions. 1,8% (CI 1,7% - 1,9%) of all reported patients with lower extremity trauma suffered from at least one nerve injury. The evaluation of PNI and control patients revealed different injury mechanisms between both groups. PNI patients were more often involved in motorcycle accidents than their counterparts (31,2% PNI versus 18,5% control group). In contrast to that only 14.4% of all PNI patients suffered from high or low falls which was exceptionally different from the control cohort with a rate of 29,8%. Consecutively the cumulative rate of traffic related injuries differed between both cohorts with 77,8% for PNI and 66,7% for trauma patients without nerve damage. The proportions of different trauma mechanisms for both groups are visualized by Fig. 1. Taken together the percentage of patients under 60 years of age and the compound proportion of traffic related injuries mainly due to motorcycle accidents were considerably higher in PNI compared to the control group. 7,9% (CI 6,2% - 9,8%) of all PNI experienced a penetrating trauma. The remaining 92,1% were affected by blunt lesions. Opposed to that only 3,2% (CI 3,0–3,3%) of the control group were afflicted by penetrating injuries leading to a proportion of 96,8% of blunt limb damages. A detailed analyses of specific lower extremity nerves (femoral, sciatic, tibial and peroneal nerve) revealed diverging patterns concerning the underlying trauma mechanism. The peroneal nerve was more frequently affected through motorcycle accidents (35,5%) than car crashes (26,8%) whereas the sciatic nerve seems to be more often impaired by car collisions (37,9%) compared to motorbike accidents (25,5%). The femoral and tibial nerves were distributed quite similar between both aforementioned categories. Furthermore pedestrian accidents presented with less sciatic (5,8%) than peroneal nerve lesions (12,7%), whereas the proportions of tibial and femoral nerve affections in this trauma category were 11,3% for tibial and 10,6% for femoral PNI. Injury rates resulting from falls exhibited similar frequencies regarding the examined nerves of the lower limb. Corresponding percentages for combined high (> 3 m) and low falls (< 3 m) ranged between a maximum of 19,3% for femoral and a minimum of 12,4% for tibial PNI. Table 2 delineates the correlation between different trauma mechanisms and particular nerve injuries of the leg. The table furthermore categorizes falls in high (> 3 m) and low falls (< 3 m).

**Table 1:** epidemiology_control_PNI

	Control group	PNI group
proportion of all leg injured patients (%)	98,2	1,8
male (%)	68,4	80,0
mean/median age (years)	46,7/46,0 (SD 21,8)	38,1/36,0 (SD 17,1)
1–15 years (%)	3,4	2,2
16–59 years (%)	67,5	86,1
60–69 years (%)	10,0	6,5
70–79 years (%)	10,3	4,1
80 years or more (%)	8,9	1,1

Table_epidemiology_control_PNI: This table shows epidemiological data of 60,422 lower extremity trauma patients with and without ascertained peripheral nerve injury. Supplementary to the mean age of both groups a further classification into 5 age bands was performed. *Control group* leg trauma patients without recorded nerve injury, *PNI group* leg trauma patients with accompanying nerve injury, *SD* standard deviation

**Table 2:** trauma mechanisms_specific nerves

	Femoral nerve	Sciatic nerve	Tibial nerve	Peroneal nerve
car (%)	25,0 (CI 16,3–36,6)	37,9 (CI 30,5–46,4)	32,0 (CI 24,5–41,0)	26,8 (CI 22,4–31,8)
motorbike (%)	31,7 (CI 21,8–44,6)	25,5 (CI 19,6–32,7)	30,9 (CI 23,6–39,8)	35,5 (CI 30,4–41,1)
bicycle (%)	4,8 (CI 1,6–11,2)	3,3 (CI 1,4–6,5)	3,1 (CI 1,1–6,7)	3,8 (CI 2,3–6,0)
pedestrian (%)	10,6 (CI 5,3–18,9)	5,8 (CI 3,1–9,7)	11,3 (CI 7,1–17,2)	12,7 (CI 9,8–16,3)
high fall (> 3 m) (%)	13,5 (CI 7,4–22,6)	12,3 (CI 8,3–17,6)	9,3 (CI 5,5–14,7)	9,9 (CI 7,3–13,1)
low fall (< 3 m) (%)	5,8 (CI 2,1–12,6)	4,5 (CI 2,3–8,1)	3,1 (CI 1,1–6,7)	3,2 (CI 1,8–5,2)
other (%)	8,7 (CI 4,0–16,4)	10,7 (CI 7,0–15,7)	10,3 (CI 6,3–15,9)	8,1 (CI 5,8–11,0)

Table_trauma mechanisms_specific nerves: This table provides detailed information about the proportional differences of underlying accident patterns leading to lower limb injuries with particular extremity nerve involvement. Truncation of numbers in a single column may cause deviations from 100%. *CI* confidence interval of 95%

### Concomitant injuries of other body segments and adjacent anatomical structures

**Fig. 2:**
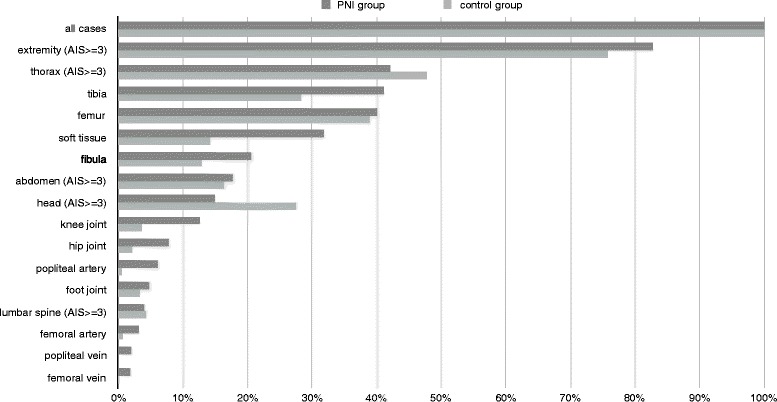
Concomitant injuries_control_PNI Appendix: Control group = leg trauma patients without recorded nerve injury; PNI group = leg trauma patients with accompanying nerve injury; AIS = abbreviated injury scale

Only significantly traumatized body regions (AIS > = 3 points) were analyzed to prevent consideration of minor lesions. PNI presented with more relevant extremity injuries than control group patients (82,7% PNI; 75,8% control). Significant abdominal involvement showed comparable proportions in both cohorts (17,8% PNI; 16,5% control), whereas accompanying considerable thorax trauma was more common in the control group (47,8%) compared to PNI patients (42,2%). The same result was observed for relevant head injuries (27,5% control; 15,0% PNI). We additionally analyzed all PNI regarding involvement of neighboring anatomical structures including bones, joints, vessels and soft tissue. The whole context is presented in Fig. 2. Traumatic affection of the tibia was seen in 41,2% of the PNI compared to 28,4% of the control patients. Damage of the fibula was also more frequently seen in patients with concomitant nerve injury (20,6% PNI; 12,9% control). In contrast to this finding femur fractures were registered almost uniformly in both groups (40,2% PNI; 39,0% control). All grades of lumbar backbone lesions were summarized and revealed almost equal rates for both cohorts (19,3% PNI; 19,1% control). Joints of the lower extremities were generally more frequently affected in the PNI cohort. 7,8% of PNI patients showed additional hip joint injury compared to 2,1% of control patients. Analysis of knee joint lesions also indicated higher rates for patients with affected peripheral nerves (12,6% PNI; 3,7% control). Foot joint damages were more often recognized in PNI (4,9%) too, but the excess was less obvious (3,4%). Extended soft tissue injury was found in 31,9% of all PNI which was more than twice as frequent as in the control group (14,2%). PNI was more numerously associated with vessel injuries compared to leg trauma without PNI. This general finding could be identified more clearly in arterial lesions, but it was also present in the rare events of vein lacerations. PNI were accompanied by femoral artery injuries in 3,2% and popliteal artery lesions in 6,2%, whereas patients of the control group infrequently exhibited femoral or popliteal artery ruptures (femoral artery 0,7%; popliteal artery 0,5%). The rates concerning vein damages are presented together with all other associated injuries in Fig. 2. We also examined distinct subsets of nerve lesions of the lower extremity regarding associated injuries of adjacent anatomical structures. A comprehensive exposition of all data is provided in Table 3. Femoral PNI was accompanied with high rates of vascular lacerations. Femoral artery (vein) injury was detected in 12,4% (5,3%) of femoral PNI, but only in 0,8% (0,2%) of all lower extremity trauma patients. Hip joint affection was seen in 6,2% of femoral PNI. In contrast to that only 2,2% of all leg injury patients exhibited hip joint lesions. Pelvic fractures were present more frequently in femoral PNI (44,2%) compared to the reference population (18,8%), whereas femur fractures revealed a similar prevalence in both groups. The rates of pelvic AIS grade 4/5 and femur fractures in sciatic PNI were within the same range of those identified in femoral PNI. Pelvic fractures were seen in 40,9% and femur fractures in 38,7% of sciatic PNI. Hip joint lesions were encountered eight times more often in sciatic PNI (17,8%) compared with the reference cohort (2,2%), while accompanying knee joint injury was registered in similar prevalence in both groups. Vascular lacerations of arterial and venous vessels were generally more common in sciatic PNI than in lower extremity trauma without sciatic lesion. This finding was even more obvious in venous injuries. Detailed information is provided in Table 3. Evaluation of tibial PNI showed a decisive association between nerve trauma and lacerations of the popliteal vessels (popliteal artery 13,7%; popliteal vein 3,9%). Non-PNI leg lesions unveiled popliteal artery (vein) ruptures only in 0,6% (0,1%). Tibial PNI also correlated with knee joint damages (12,3% tibial PNI; 3,8% all patients), but rates of foot joint injuries were equivalent in both cohorts (3,4%). The proportion of tibial PNI associated femur fractures was in line with those of other PNI subsets (42,2%), whereas fractures of the tibia (45,1%) and fibula (22,5%) were more regularly seen in the tibial PNI cohort than in the reference group (tibia 28,6%; fibula 13,1%). Tibial (fibular) fractures and knee (foot) joint dislocations were even more frequently seen in peroneal compared with tibial PNI (peroneal PNI: tibial fracture 55,0%; fibular fracture 28,4%; knee joint dislocation 19,0%; foot joint dislocation 6,5%). Popliteal vessel damages were identified in 6,7% (popliteal artery) and 2,0% (popliteal vein) of peroneal PNI which was less common than in leg trauma patients with concomitant tibial nerve injuries.

**Table 3:** Concomitant injuries_specific nerves

Concomitant injury	Femoral nerve	Sciatic nerve	Tibial nerve	Peroneal nerve	All patiens with leg trauma
pelvis (AIS grade 4/5) (%)	44,2 (CI 32,8–58,3)	40,9 (CI 33,6–49,3)	–	–	18,8 (CI 18,5–19,2)
femur (%)	41,6 (CI 30,6–55,3)	38,7 (CI 31,6–46,9)	42,2 (CI 33,7–52,1)	–	39,0 (CI 38,5–39,5)
tibia (%)	–	–	45,1 (CI 36,4–55,3)	55,0 (CI 48,9–61,7)	28,6 (CI 28,2–29,0)
fibula (%)	–	–	22,5 (CI 16,5–30,1)	28,4 (CI 24,1–33,3)	13,1 (CI 12,8–13,4)
hip joint (%)	6,2 (CI 2,5–12,8)	17,8 (CI 13,2–23,7)	–	–	2,2 (CI 2,1–2,3)
knee joint (%)	–	4,5 (CI 2,3–7,8)	12,3 (CI 7,9–18,1)	19,0 (CI 15,5–23,0)	3,8 (CI 3,7–4,0)
foot joint (%)	–	–	3,4 (CI 1,4–7,1)	6,5 (CI 4,5–9,0)	3,4 (CI 3,3–3,6)
femoral artery (%)	12,4 (CI 6,8–20,8)	2,2 (CI 0,8–4,9)	–	–	0,8 (CI 0,7–0,8)
popliteal artery (%)	–	3,7 (CI 1,8–6,8)	13,7 (CI 9,1–19,8)	6,7 (4,7–9,3)	0,6 (CI 0,6–0,7)
femoral vein (%)	5,3 (CI 1,9–11,6)	3,3 (CI 1,5–6,4)	–	–	0,2 (CI 0,2–0,2)
popliteal vein (%)	–	1,9 (CI 0,6–4,3)	3,9 (CI 1,7–7,7)	2,0 (1,0–3,7)	0,1 (CI 0,1–0,1)

Table_ concomitant injuries_specific nerves: This table shows frequencies of accompanying bone, joint and vessel injuries for lower extremity trauma patients with distinct nerve lesions (113 femoral, 269 sciatic, 204 tibial, 538 peroneal). The column on the right side reports the overall incidence of associated injuries for 60,422 leg trauma patients

*AIS* abbreviated injury scale, *CI* confidence interval of 95%

### Severity of injury and outcome

Leg injured patients with and without concomitant PNI were evaluated for trauma severitiy utilizing the ISS and outcome measured by the need for further rehabilitation after hospital discharge and GOS. We excluded all lower limb trauma patients from further analysis which died during the period of primary hospital care (11,8% of all cases) to rule out a possible bias resulting from deficient data regarding nerve integrity within this population. Both groups presented with comparable mean ISS scores (22,7 PNI vs. 22,1 control) and achieved similar rates of good recovery defined by GOS scores of 4 or 5 (89,6% PNI vs. 88,8% control), but a significant difference between both cohorts was found in terms of the amount of patients requiring further inpatient rehabilitation (45,4% PNI vs. 34,3% control). An overview of injury severity and outcome parameters is given in Table 4.

**Table 4:** Severity of injury_outcome_control_PNI

	control group	PNI group
mean/median ISS	22,1/19,0 (SD 11,3)	22,7/20,0 (SD 11,2)
ICU treatment (%)	86,5 (CI 85,7–87,2)	95,5 (CI 89,7–100,0)
mean/median hospital stay (days)	28,2/22,0 (SD 23,3)	40,5/33,0 (SD 29,0)
GOS 2/3 (%)	11,2 (CI 10,9–11,5)	10,4 (CI 8,5–12,6)
GOS 4/5 (%)	88,8 (CI 87,9–89,6)	89,6 (CI 83,9–95,6)
discharge_home (%)	48,8 (CI 48,2–49,4)	39,5 (CI 35,8–43,5)
discharge_rehabilitation (%)	34,3 (CI 33,8–34,8)	45,4 (CI 41,4–49,6)
discharge_hospital (%)	13,0 (CI 12,7–13,4)	11,5 (CI 9,6–13,8)
discharge_other (%)	3,9 (CI 3,7–4,0)	3,6 (CI 2,6–5,0)

Table_severity of injury_outcome_control_PNI: Lower extremity trauma cases with peripheral nerve injury (PNI group) are compared with leg trauma cases without recorded nerve damage (control group) regarding severity of injury and distinctive outcome variables

*ISS* Injury Severity Score, *ICU* Intensive Care Unit, *GOS* Glasgow Outcome Scale, *SD* standard deviation, *CI* confidence interval of 95%

### Treatment

67,0% of PNI and 68,5% of control patients were treated at tertiary care centers which implicates no significant differences between both groups regarding infrastructural aspects like availability of equipment and medical specialists. 95,5% of the PNI compared to 86,5% of the control group were attended to the ICU altogether indicating more severely affected trauma victims in the PNI cohort. Patients with injured extremity nerves had a distinctively prolonged inpatient hospital stay compared to those individuals with leg trauma showing intact neural structures with 40,3 days mean (33,0 days median) for PNI versus 25,8 days mean (20,0 days median) for those belonging to the control group. Six hundred fifty of 1058 PNI patients were registered by the standard documentation sheet of the TR-DGU which contains sufficient data about invasive therapies. Unfortunately the reduced set was used in the remaining PNI which does not comprise any information about surgical procedures. 16,5% of the PNI patients with standard documentation received nerve specific surgery (e.g. decompression, nerve suturing or grafting) during the primary hospital stay. The intervention rate of the individual lower extremity nerves revealed a surpassing frequency of procedures for the peroneal nerve (20,9%), whereas the operation rates of the femoral, sciatic and tibial nerve injuries presented homogeneously lower. Detailed data are shown in Table 5.

**Table 5:** Operation rate_specific nerves

	Operation rate (%)
all patients with nerve injuries (*n* = 650)^a^	16,5 (CI 13,5–19,9)
femoral nerve injuries (*n* = 66)^a^	15,2 (CI 7,3–27,9)
sciatic nerve injuries (*n* = 162)^a^	13,6 (CI 8,5–20,6)
tibial nerve injuries (*n* = 168)^a^	14,3 (CI 9,2–21,3)
peroneal nerve injuries (*n* = 292)^a^	20,9 (CI 16,0–26,8)
toe nerve injuries (*n* = 8)^a^	0.0 (CI 0,0–46,1)

Table_operation rate_specific nerves: This table shows the operation rates of 650 patients with injured nerves of the lower extremity

*CI* confidence interval of 95%

^a^Some patients may suffer from more than one nerve damage

## Discussion

The purpose of this study was to elucidate epidemiological patterns and characteristics of lower extremity nerve trauma in Central Europe by retrospective analysis of 60,422 leg injured patients entered into the TR-DGU database between 2002 and 2015. This investigation suggests concomitant PNI in 1,8% of patients suffering from lower extremity injury. Physicians may attend vital threats at first in situations with life threatening polytrauma or head injury which could possibly lead to underreporting of extremity lesions including nerve damage [7], but after primary stabilization of vital functions a detailed secondary survey including nerve function is mandatory according to the TR-DGU protocol which may reduce diagnostic failure. Our findings are in line with the results of other studies from various regional and socioeconomic settings which referred nerve lesion rates between 1 and 3% of all patients admitted to trauma hospitals [3, 8–11], whereas an investigation of children suffering from traumatic brain injury revealed electrodiagnostically confirmed peripheral nerve damage in 7% of cases [12]. There was a predominant proportion of male PNI patients in this study (80%) which is congruent with the majority of previously performed trials [2, 3, 8, 11, 13–15]**,** but there are also exceptions with almost equal distributions between both sexes [16]. On the average PNI patients in our study were significantly younger compared to the control group (38,1 years vs. 46,7 years). This correlation agrees with the findings of the retrospective cross-sectional study performed by Saadat et al., where PNI trauma patients revealed a significant lower mean age than their counterparts without nerve damage (PNI 28,6 years; non-PNI trauma 33 years) [3]. Eser et al. refer a mean age of 31,8 years for 938 patients with PNI [14]. Generally younger patients are considered to be more vulnerable regarding nerve injury [11, 17, 18]. Our research suggests peroneal (51%) and sciatic nerves (25%) being the most frequently affected neural structures in lower extremity trauma which is supported by other trauma studies and the UVG database [9, 18, 19]. The more superficial anatomical position of the peroneal nerve could eventually lead to a surpassing trauma rate, whereas the femoral and tibial nerves may take advantage of a beneficial anatomical course [20]. A Mexican single-center series and a cross sectional study of earthquake victims exposed the sciatic nerve being the most frequently injured major nerve of the lower limb [11, 17]. In our study patients with PNI revealed a mean ISS of 22,7 compared to an average ISS of 22,1 in the control group. These results are in harmony with the mean ISS of 23,1 noticed in 162 PNI patients by Noble and colleagues, but they contradict the findings of Saadat et al. who reported lower scores for PNI trauma patients (ISS 5,5) and their counterparts without nerve involvement (ISS 7,1) [3, 8]. Only patients showing at least one lesion with an AIS of 3 or higher were included into this investigation according to the predefined inclusion criterion which could possibly contribute to generally increased ISS compared to the results of the aforementioned study. Registered ISS of this study were slightly inferior to those of pedestrian traffic collision victims (ISS 26,2) and motor vehicle passengers (ISS 25,4) in a study performed by Reith and colleagues [21]. This study suggests higher proportions of bone fractures, joint dislocations and vessel ruptures for PNI compared to the control group, as presented by Fig. 2. Garozzo et al. report on 72 patients with lumbosacral plexus lesions who suffered from additional bone damages in 83% and vascular lesions in 8% [15]. Kim et al. describe fractures to be the cause of sciatic nerve injuries in 17%, peroneal nerve lesions in 7% and tibial nerve damages in 41% in their surgical series [22–24]. About one out of three patients with peroneal dysfunction following knee dislocation or traumatic sciatic neurotmesis sustain concomitant vascular injuries [25, 26]. Levy et al. report on the strong linkage between PNI and orthopaedic trauma including fractures in patients undergoing skiing and snowboarding accidents [27]. The PNI patients of our series had less additional head injuries than their counterparts (PNI 15%; control 27,5%). This outcome is distinguished from the results of Noble et al., which mention head injury rates of 60% in a PNI population [8]. The difference may be explained by our limited focus on PNI patients with lower extremity trauma. Data analysis from the TR- DGU highlights different injury mechanisms for both investigated cohorts. Leg trauma without nerve damage is more commonly associated with falls (control group 29,8%; PNI group 14,4%), whereas nerve injury seems to be more often a consequence of motorcycle accidents (control group 18,5%; PNI group 31,2%) and penetrating trauma (control group 3,2%; PNI group 7,9%). The correlation between penetration and nerve injury is supported by other studies dealing with PNI [3, 28]. 60% (21%) of lumbosacral plexus lesions were categorized as consequences of car crashes (motorbike accidents) by Garozzo et al., whereas only 6% of these injuries were due to falls [15]. Uzun et al. also report on traffic accidents as the main cause of nerve injuries [29]. Another study denominates nerve trauma as a result of motor vehicle collisions in 39,7% and gunshot wounds in 32,4% which underlines the significance of environmental factors regarding PNI [13]. PNI and control patients were characterized by similar ISS scores which suggests a comparable overall trauma severity for both cohorts. This creates a common basis concerning consecutive outcome measurement. The analyzed database provides information about GOS, duration of hospital stay and need for further rehabilitation therapy after discharge. GOS was analogous in both groups, but this score alone could be considered as an inappropriate parameter for comparison because of its intrinsic nature as an outcome measure after brain damage [30]. However, the majority of cases in our study did not sustain a severe head injury. Good outcome represented by GOS grade 4 or 5 signifies physical independence regarding activities of daily living which may be reached by plenty of patients with PNI despite of their burden caused by neuroma associated pain, stress and diminished health related quality of life [4, 6]. Intiso et al. found comparable good functional recoveries after intensive rehabilitation for multiply injured patients with and without PNI measured by Barthel and mRS scores [31]. More suitable comparative parameters could be the duration of hospital stay and the need for further rehabilitation after discharge. PNI may lead to immobilization promoting prolonged inpatient stay and increased rehabilitation treatment rate which causes extended periods of absenteeism with associated detrimental economical and social consequences. In our study both parameters were distinctly increased within the PNI group which is an important finding for the individual patient and also for the healthcare system. Our results are congruent with the swiss accident statistics database analysis from 1997 and other series which identified nerve trauma as a costly lesion associated with a higher risk for loss of productivity compared to injuries without nerve damage [31–34]. Besides of the merits of this multi-centre study concerning standardized evaluation of a large number of patients comprising all age groups from different European countries the main limitation results from its retrospective character. The TR-DGU primary focuses on major trauma management which could eventually contribute to a significant underreporting of non life-threatening injuries. Therefore our presented data concerning PNI incidence rates should preferably be regarded as lower limit values for patients with significant leg injuries. Additionally total PNI incidence rates cannot be extracted from the TR-DGU database, because minor PNI lesions without admission to hospital via emergency room are beyond the scope of this registry.

## Conclusions

Peripheral nerve injuries apparently affect about 1,8% of patients with significant lower limb trauma. In this study the most frequently afflicted neural structure was the peroneal nerve. Our study reveals an increased likelihood for young male patients to suffer from nerve involvement. The dimension of global injury severity in leg trauma patients appears to be independent of an additional nerve damage, but individuals with involvement of the peripheral nervous system generally show a different pattern of accompanying lesions, an extended inpatient stay and a higher need for subsequent rehabilitation. The most common causes of PNI in this central European based study were motorbike and car accidents, whereas lower extremity trauma without nerve damage seems to be mainly associated with car accidents and falls.

## Abbreviations

AIS: Abbreviated Injury Scale (version 2005 update 2008)
CI: 95% confidence interval
GOS: Glasgow Outcome Scale
ICU: Intensive Care Unit
ISS: Injury Severity Score
mRS: Modified Rankin Scale
PNI: Peripheral nerve injury
TR-DGU: TraumaRegister DGU®
UVG: Swiss accident insurance

## References

[1] Portincasa A, Gozzo G, Parisi D, Annacontini L, Campanale A, Basso G (2007). Microsurgical treatment of injury to peripheral nerves in upper and lower limbs: a critical review of the last 8 years. Microsurgery.

[2] Stone L, Keenan MA (1988). Peripheral nerve injuries in the adult with traumatic brain injury. Clin Orthop Relat Res.

[3] Saadat S, Eslami V, Rahimi-Movaghar V (2011). The incidence of peripheral nerve injury in trauma patients in Iran. Turk J Trauma Emerg Surg.

[4] Ciaramitaro P, Mauro Mondelli M, Logullo F, Grimaldi S, Battiston B, Sard A, on behalf of the Italian network for traumatic neuropathies (2010). Traumatic peripheral nerve injuries: epidemiological findings, neuropathic pain and quality of life in 158 patients. J Peripher Nerv Syst.

[5] Kretschmer T, Antoniadis G, Assmus H. Nervenchirurgie: trauma, tumor, Kompression, Chapter 4. Berlin and Heidelberg: Springer; 2014.

[6] Wojtkiewicz DM, Saunders J, Domeshek L, Novak CB, Kaskutas V, Mackinnon SE (2015). Social impact of peripheral nerve injuries. Hand.

[7] Kushwaha VP, Garland DG (1998). Extremity fractures in the patient with a traumatic brain injury. J Am Acad Orthop Surg.

[8] Noble J, Munro CA, Prasad VS, Midha R (1998). Analysis of upper and lower extremity peripheral nerve injuries in a population of patients with multiple injuries. J Trauma.

[9] Robinson LR (2000). Traumatic injury to peripheral nerves. Muscle Nerve.

[10] Robinson LR (2004). Traumatic injury to peripheral nerves. Suppl Clin Neurophysiol.

[11] Castillo-Galvan ML, Martinez-Ruiz FM, Garza-Castro O, Elizondo- Omana RE, Guzman- Lopez S (2014). Study of peripheral nerve injury in trauma patients. Gaceta Medica de Mexico.

[12] Philip PA, Philip M (1992). Peripheral nerve injuries in children with traumatic brain injury. Brain Inj.

[13] Wee AS, Truitt NR, Smith LD (2006). Type and frequency of peripheral nerve injuries encountered in a clinical neurophysiology laboratory. J Miss State Med Assoc.

[14] Eser F, Aktekin LA, Bodur H, Atan C (2009). Etiological factors of traumatic peripheral nerve injuries. Neurol India.

[15] Garozzo D, Zollino G, Ferraresi S (2014). In lumbosacral plexus injuries can we identify indicators that predict spontaneous recovery or the need for surgical treatment? Results from a clinical study on 72 patients. J Brachial Plex Peripher Nerve Inj.

[16] Taylor CA, Braza D, Rice JB, Dillingham T (2008). The incidence of peripheral nerve injury in extremity trauma. Am J Phys Med Rehabil.

[17] Ahrari MN, Zangiabadi N, Asadi A, Sarafi Nejad A (2006). Prevalence and distribution of peripheral nerve injuries in victims of bam earthquake. Electromyogr Clin Neurophysiol.

[18] UVG https://www.unfallstatistik.ch.

[19] Kouyoumdjian JA (2006). Peripheral nerve injuries: a retrospective survey of 456 cases. Muscle Nerve.

[20] Immerman I, Price AE, Alfonso I, Grossman JAI (2014). Lower extremity nerve trauma. Bull Hosp Joint Dis.

[21] Reith G, Lefering R, Wafaisade A, Hensel KO, Paffrath T, Bouillon B, Probst C, TraumaRegister DGU (2015). Injury pattern, outcome and characteristics of severely injured pedestrian. Scand J Trauma Resusc Emerg Med.

[22] Kim DH, Cho Y, Ryu S, Tiel RL, Kline DG (2003). Surgical management and results of 135 tibial nerve lesions at the Louisiana State University health sciences center. Neurosurgery.

[23] Kim DH, Murovic JA, Tiel RL, Kline DG (2004). Management and outcomes in 318 operative common peroneal nerve lesions at the Louisiana State University health sciences center. Neurosurgery.

[24] Kim DH, Murovic JA, Tiel R, Kline DG (2004). Management and outcomes in 353 surgically treated sciatic nerve lesions. J Neurosurg.

[25] Vayvada H, Demirdöver C, Menderes A, Yilmaz M, Karaca C (2013). The functional results of acute nerve grafting in traumatic sciatic nerve injuries. Turk J Trauma Emerg Surg.

[26] Krych AJ, Giuseffi SA, Kuzma SA, Stuart MJ, Levy BA (2014). Is peroneal nerve injury associated with worse function after knee dislocation?. Clin Orthop Relat Res.

[27] Levy AS, Smith RH (2000). Neurologic injuries in skiers and snowboarders. Semin Neurol.

[28] Babar SM (1993). Peripheral nerve injuries in a third world country. Cent Afr J Med.

[29] Uzun N, Tanriverdi T, Savrun FK (2006). Traumatic peripheral nerve injuries : demographic and electrophysiological findings of 802 patients from a developing country. J Clin Neuromuscul Dis.

[30] Jennett B, Bond M (1975). Assessment of outcome after severe brain damage. Lancet.

[31] Intiso D, Grimaldi G, Russo M, Maruzzi G, Basciani M, Fiore P, Zarrelli M, Di Rienzo F (2010). Functional outcome and health status of injured patients with peripheral nerve lesions. Injury.

[32] AWMF 005/010 S3 Leitlinie: Versorgung peripherer Nervenverletzungen (06/2013). http://www.awmf.org.

[33] Rosberg HE, Carlsson KS, Höjgard S, Lindgren B, Lundborg G, Dahlin LB (2005). Injury to the human median and ulnar nerves in the forearm - analysis of costs for treatment and rehabilitation of 69 patients in southern Sweden. J Hand Surg Br.

[34] Lad SP, Nathan JK, Schubert RD, Boakye M (2010). Trends in median, ulnar, radial, and brachioplexus nerve injuries in the United States. Neurosurgery.

